# Effects of hypoxia and reoxygenation on mitochondrial functions and transcriptional profiles of isolated brain and muscle porcine cells

**DOI:** 10.1038/s41598-022-24386-0

**Published:** 2022-11-18

**Authors:** Linda Adzigbli, Eugene P. Sokolov, Klaus Wimmers, Inna M. Sokolova, Siriluck Ponsuksili

**Affiliations:** 1 Research Institute for Farm Animal Biology (FBN), Institute of Genome Biology, Dummerstorf, Germany; 2 Department of Marine Biology, Institute for Biological Sciences, University of Rostock, Rostock, Germany; 3 Leibniz Institute for Baltic Sea Research, Leibniz Science Campus Phosphorus Research, Warnemünde, Rostock, Germany; 4 Department of Maritime Systems, Interdisciplinary Faculty, University of Rostock, Rostock, Germany

**Keywords:** Transcriptomics, Zoology, Physiology, Metabolism, Respiration

## Abstract

Oxygen fluctuations might occur in mammalian tissues under physiological (e.g. at high altitudes) or pathological (e.g. ischemia–reperfusion) conditions. Mitochondria are the key target and potential amplifiers of hypoxia-reoxygenation (H-R) stress. Understanding the mitochondrial responses to H-R stress is important for identifying adaptive mechanisms and potential therapeutic solutions for pathologies associated with oxygen fluctuations. We explored metabolic response to H-R stress in two tissue types (muscle and brain) with different degrees of hypoxia tolerance in a domestic pig *Sus scrofa* focusing on the cellular responses independent of the systemic regulatory mechanisms. Isolated cells from the skeletal muscle (masseter) and brain (thalamus) were exposed to acute short-term (15 min) hypoxia followed by reoxygenation. The mitochondrial oxygen consumption, reactive oxygen species (ROS) production rates and transcriptional profiles of hypoxia-responsive mRNA and miRNA were determined. Mitochondria of the porcine brain cells showed a decrease in the resting respiration and ATP synthesis capacity whereas the mitochondria from the muscle cells showed robust respiration and less susceptibility to H-R stress. ROS production was not affected by the short-term H-R stress in the brain or muscle cells. Transcriptionally, prolyl hydroxylase domain protein EGLN3 was upregulated during hypoxia and suppressed during reoxygenation in porcine muscle cells. The decline in EGLN3 mRNA during reoxygenation was accompanied by an upregulation of hypoxia-inducible factor subunit α (HIF1A*)* transcripts in the muscle cells. However, in the brain cells, HIF1A mRNA levels were suppressed during reoxygenation. Other functionally important transcripts and miRNAs involved in antioxidant response, apoptosis, inflammation, and substrate oxidation were also differentially expressed between the muscle and brain cells. Suppression of miRNA levels during acute intermittent hypoxia was stronger in the brain cells affecting ~ 55% of all studied miRNA transcripts than in the muscle cells (~ 25% of miRNA) signifying transcriptional derepression of the respective mRNA targets. Our study provides insights into the potential molecular and physiological mechanisms contributing to different hypoxia sensitivity of the studied tissues and can serve as a starting point to better understand the biological processes associated with hypoxia stress, e.g. during ischemia and reperfusion.

## Introduction

Oxygen is essential for the survival of animals owing to its role in aerobic ATP production in the mitochondria. In most terrestrial habitats (except underground or at high altitudes), ambient oxygen levels are not limiting for aerobic metabolism, and hypoxic episodes are rare and transient followed by reoxygenation. However, low oxygen levels might occur in the tissue when oxygen demand exceeds oxygen supply (e.g. in exercising muscle) or under pathological conditions that restrict oxygen delivery to the tissue (ischemia)^1,2^. Hypoxia-reoxygenation (H-R) stress can severely damage organs and tissues as shown in human pathologies caused by heart attack, stroke, respiratory failure, sleep apnea, surgery, or organ transplantation^3–6^. There is considerable variability in the tolerance to oxygen fluctuations between different organs, tissues and different species of animals^7^. The mechanisms underlying this variability are not yet fully understood but likely involve variation in the expression of protective mechanisms that support ATP production and prevent mitochondrial injury^8–10^.

Mitochondria are important targets and potential amplifiers of H-R stress in animal cells. Insufficient oxygen supply suppresses ATP generation via oxidative phosphorylation (OXPHOS) and can result in energy deficiency^11,12^. Furthermore, hypoxia can lead to the overproduction of reactive oxygen species (ROS) and calcium overload in the mitochondria^13^. Paradoxically, reoxygenation can amplify mitochondrial stress and is a major source of tissue damage during oxygen fluctuations. Reactivation of mitochondrial OXPHOS results in a burst in ROS production as a result of electron leak from the highly reduced metabolic intermediates and reverse electron transport^11,14^. Excessive ROS levels might result in oxidative injury to the proteins, DNA and membrane lipids, suppress mitochondrial OXPHOS capacity and impair the recovery of energy homeostasis in the cell^3,6,8^. In extreme cases, mitochondrial damage can lead to metabolic collapse, the release of cytochrome c and initiation of the apoptotic cascade resulting in tissue damage and organ failure^11,15^. Studies of the species evolutionarily adapted to hypoxic environments (such as intertidal invertebrates, anoxia-tolerant fish, reptiles and naked mole rats) indicate that tolerance to H-R is associated with the ability to maintain high mitochondrial respiratory flux and mitigate oxidative stress during oxygen fluctuations^1,7,16,17^. To date, most studies of mitochondrial respiration and ROS production during oxygen fluctuations involve comparisons of species with different degrees of hypoxia tolerance^7,16,17^. The direct comparisons of the mitochondrial responses between tissues with different degree of hypoxia tolerance within the same organisms are, to the best of our knowledge, unavailable.

Multiple molecular and cellular mechanisms have evolved to sense oxygen and protect against stress induced by oxygen fluctuations. An essential regulator of hypoxia response in animals is the hypoxia-inducible factor transcription factor HIF-1^18^.The HIF-1α subunit is continually synthesized but rapidly degraded in the presence of oxygen^19^. Under hypoxic conditions, HIF-1α accumulates and binds with the constitutive HIF-1β subunit to form an active HIF-1 that initiates a transcriptional cascade regulating both oxygen supply and oxygen demand of the cell^20,21^. HIF-1-dependent cascade involves target genes associated with angiogenesis, energy metabolism, redox homeostasis, cell proliferation, autophagy and apoptosis^19,22^^]^. A major group of HIF-1 targets includes genes involved in metabolic rewiring and the shift of cellular metabolism from aerobic OXPHOS to anaerobic glycolysis to meet energy demands under low oxygen conditions^19,23,24^. HIF-1α also regulates mitochondrial respiratory capacity limiting ROS production, regulating cytochrome oxidase subunit expression and suppressing metabolite entry into the tricarboxylic acid (TCA) cycle^25,26^.

In recent years, microRNAs (miRNAs) have emerged as important regulators of hypoxic response in animals^27,28^. Small non-coding miRNAs regulate gene expression by interacting with the 3′ untranslated region of target mRNAs to induce mRNA degradation and translational repression. Currently, over 90 hypoxia-inducible miRNAs (hypoxamiRs) have been identified in mammals regulating angiogenesis, cell survival and metabolism^29^. Many hypoxamiRs including a master regulator of hypoxia response miR-210 are transcriptionally controlled by HIF-1 ensuring coordination of the transcriptional upregulation and gene silencing in hypoxia^28,30^. Several hypoxamiRs directly regulate the mitochondrial function and thus might play an important role in the mitochondrial responses to hypoxia^31,32^. This includes the regulation of mRNA levels of OXPHOS-related proteins by miR-338, miR181c and miR-210^29,33,34^, mitochondrial fatty acid oxidation (miR-214 and miR-27b) and biogenesis (miR27b, miR25 and miR-696)^29,35,36^.

Our study aims at gaining insight into the mammalian metabolic stress response to H-R by investigating the effects of acute short-term hypoxia and reoxygenation on functional mitochondrial responses (oxygen consumption and ROS production) and transcriptional changes in mRNA and miRNA profiles of the skeletal muscle and brain cells of the pig *Sus scrofa*. The two tissues were chosen based on their different dependence on aerobic metabolism and hypoxia tolerance. The mammalian brain is an organ with high dependence on oxygen availability, low glycolytic capacity and low hypoxia tolerance^37^. In this study, we focused on the thalamus as one of the most hypoxia-sensitive brain regions^38^. Unlike the brain, the skeletal muscle tissue is well adapted to hypoxic conditions owing to its metabolic and structural plasticity^39^. The skeletal muscle might experience hypoxia under physiological conditions such as vigorous physical exercise and can sustain on anaerobic glycolysis^40^. Here we focused on the masseter muscle from the masticatory system of pigs that consists of ~ 75% type IIA-fibers with high myosin ATPase activity and high oxidative and glycolytic capacity^41,42^. We hypothesized that the cells from the more hypoxia-tolerant tissue (masseter) will maintain stable respiration and ROS production during acute hypoxia-reoxygenation stress, whereas the more hypoxia-sensitive brain cells might respond with a decrease in respiration and elevated ROS production indicating mitochondrial damage. We also hypothesized that the transcriptional and miRNA response to H-R will differ between the two cell types, with a stronger upregulation of glycolysis and protective mechanisms (such as antioxidants, apoptosis, inflammation, angiogenesis, or mitochondrial fusion) in the more hypoxia-tolerant muscle cells. To test these hypotheses, we isolated cells from the muscle and brain tissues and conducted an in vitro H-R exposure (15 min hypoxia and 10 min of reoxygenation) for determination of mitochondrial functional traits. We also analyzed the expression of 88 hypoxia-sensitive mRNAs and 43 miRNA in isolated cells exposed to normoxia, hypoxia and post-hypoxic recovery focusing on the HIF signalling, mitochondrial OXPHOS, glycolysis, apoptosis, autophagy and inflammation pathways^19^.

## Materials and methods

### Animal care and tissue sampling

Animal care and tissue collection procedures were approved by the Animal Care Committee of the Institute for Farm Animal Biology and followed the approved guidelines for good scientific practice by the European Communities Council Directive of 24 November 1986 (86/609/EEC). All experimental procedures are reported according to the ARRIVE guidelines^43^. All the necessary and required measures were taken to minimize pain and discomfort. The animals were used for meat production and underwent no experimental treatment, diagnostic sampling or other intervention. Therefore, a specific ethical approval was not required. Animal handling and humane killing were in accordance with the applicable ethical laws, guidelines and provisions. In total, 14 female pigs with an average age of 175 ± 11 days and an average mass of 105 ± 9 kg were used. Eight pigs were used for the respiration measurements and six for mRNA and miRNA studies. The muscle (masseter) and brain (thalamus) tissues were dissected. The subsamples of each tissue were immediately frozen in the liquid nitrogen and stored at − 80 °C for mRNA and miRNA analyses. The remaining tissue was used for cell isolation, and the isolated cells were exposed to H-R in vitro. Oxygen consumption and expression of the target mRNA and miRNA were measured in the isolated cells exposed to normoxia, hypoxia and post-hypoxic recovery. The normoxic cells were collected directly after cell isolation. The hypoxic and recovering cells were collected from the respirometer chamber after exposure to hypoxia and reoxygenation, respectively (see below “Oxygen consumption rate (ṀO_2_) and ROS measurements”).

### Cell isolation

The muscle and brain tissues (~ 1 g) were separately placed on a plastic Petri dish containing 3–4 volumes of ice-cold biopsy preservation solution BIOPS (50 mM K-MES, 20 mM taurine, 0.5 mM dithiothreitol, 6.56 mM MgCl_2_, 5.77 mM adenosine triphosphate (ATP), 15 mM phosphocreatine, 20 mM imidazole, 10 mM Ca-ethylene glycol-bis(2-aminoethylether)-N,N,N,N-tetraacetic acid (EGTA) buffer, 0.1 µM free calcium, pH 7.1), transferred to the laboratory on ice and processed within 10 min of collection. Tissues were sliced into strips (2–5 mm long and about 1 mm in diameter, 5–7 mg wet mass) and digested for 10–15 min in 0.05% trypsin solution with gentle shaking at room temperature. The digestion was stopped by adding 5–10% fetal calf serum. The cell suspension was diluted with phosphate buffered saline (PBS) and centrifuged at 200 × g for 5 min at 25 °C. The cell pellets were washed twice with PBS and resuspended in 0.5–1 ml of fresh PBS. The cell suspensions of the masseter mostly contained myocytes, whereas thalamus isolates were likely heterogeneous (relay cells, interneurons and thalamic reticular nucleus cells)^44^. However, because the entire thalamus was used in all isolations, the relative composition of the cell isolates from different animals is expected to be similar.

### Oxygen consumption rate (ṀO_2_) and ROS measurements

Oxygen consumption and ROS production were measured in isolated permeabilized skeletal muscle and brain cells at 37 °C using an Oxygraph 2 k high-resolution respirometer (Oroboros, Innsbruck, Austria) and an integrated DatLab 6 software^45^. Oxygen consumption was measured using a Clark-type electrode calibrated with 100% (air-saturated assay buffer) and 0% (saturated solution of sodium dithionite) solutions. After the oxygen signal stabilized (background flux of ± 1 pmol O_2_ s^−1^ ml^−1^), cell suspensions were added to the respiration chambers containing 2 ml of temperature-equilibrated MiR05 solution (110 mM sucrose, 60 mM K lactobionate, 0.5 mM EGTA, 3 mM MgCl_2_, 20 mM taurine, 10 mM KH_2_PO_4_, 20 mM 2-[4-(2-hydroxyethyl)piperazin-1-yl]ethanesulfonic acid (HEPES), 1 g l^-1^ essentially fatty acid free bovine serum albumin (BSA), pH 7.1). The cells were permeabilized by addition of saponin (25 μM) to the respiration chamber. Two independent substrate-uncoupler inhibitor titrations (SUITs) were conducted to measure the effect of H-R stress on respiration of the resting (LEAK) and phosphorylating (OXPHOS) mitochondria (SUIT1) and on cytochrome c oxidase (COX) activity (SUIT2). The effect of H-R stress on LEAK and OXPHOS was measured in SUIT1 with the following sequential additions to the same assay media: 1) 5 mM pyruvate with 2 mM malate to spark Complex I (NADH-linked) respiration (LEAK I; state 2); 2) 10 mM succinate to additionally stimulate the electron flow through Complex II (LEAK I + II; state 2); 3) 2.5 mM ADP to achieve ADP-stimulated OXPHOS state (state 3); 4) 5 mM cytochrome c as quality control to check the intactness of the mitochondrial membrane. An increase in the mitochondrial respiration due to cytochrome c addition was < 5% indicating integrity of mitochondria (data not shown). The permeabilized cells were then exposed to hypoxia (~ 0% O_2_) by maintaining ADP-stimulated respiration until the oxygen in the chamber was depleted. The cells were maintained in hypoxia for 15 min, after which the oxygen tension was raised and mitochondria allowed to recover for 10 min (reoxygenation). After reoxygenation, post-hypoxic OXPHOS respiration rate was recorded in the assay containing Complex I and II substrates (OXPHOSreox) and the SUIT1 continued as follows: 1) 2.5 μM oligomycin to inhibit mitochondrial F_O_, F_1_-ATPase and measure post-hypoxic LEAK respiration (LEAKI + IIreox, state 4); 2) 1 μM rotenone to inhibit the electron flux through Complex I and determine LEAKIIreox (state 4); 3) 7.5 mM carbonyl cyanide m-chlorophenyl hydrazone (CCCP) to uncouple the mitochondrial electron transport system (ETS) (ETSreox); 4) 2.5 μM antimycin A to inhibit electron flux through Complex III; 5); 6) 40 mM KCN to measure non-mitochondrial respiration (< 10% of the total oxygen consumption rate, data not shown).

SUIT2 to measure the effect of H-R stress on COX involved the following sequential titration steps: 1) 7.5 mM carbonyl cyanide m-chlorophenyl hydrazone (CCCP) to uncouple the mitochondrial ETS; 2) 2.5 μM antimycin A to inhibit electron flux through Complex III; 3) 0.5 mM N,N,N΄,N΄,-tetramethyl-p-phenylenediamin (TMPD) and 2 mM ascorbate to stimulate the activity of Complex IV (COX). The permeabilized cells were then exposed to H-R stress as described above. Pre- and post-hypoxia COX activity was recorded, and 40 mM KCN was used to measure non-mitochondrial respiration (< 10% of the total oxygen consumption rate, data not shown).

Efflux of H_2_O_2_ was measured simultaneously with ṀO_2_ using Fluorescence-Sensor Green (525 nm) integrated with Oxygraph 2 k in an assay buffer containing 10 μM Amplex UltraRed, 1 U ml^-1^ horseradish peroxidase and 5 U ml^-1^ superoxide dismutase (SOD)^45^. A two-step calibration was conducted with 0.1 μM H_2_O_2_ before and after the addition of the mitochondrial suspension. ROS production was measured as the rate of H_2_O_2_ efflux in LEAK and OXPHOS states and corrected for the baseline measured in the absence of cells.

Protein concentrations in the isolated cells were measured using a Bio-Rad Bradford protein assay (Bio-Rad, Hercules, CA, USA)^46^ using BSA as a standard. Protein concentrations of the cell suspensions were corrected for the BSA content of the assay media. The cellular protein content in the respirometry chambers (assay volume 2.1 ml) was ~ 50–500 µg ml^−1^, depending on the isolation. Respiration rates and ROS production were expressed as nmol O_2_ min^−1^ mg^−1^ cellular protein and nmol H_2_O_2_ min^−1^ mg^−1^ cellular protein, respectively. Trypan Blue exclusion assay did not reveal any loss of cell viability after H-R stress in the muscle or brain cell isolates (data not shown) reflecting short H-R exposure times.

Mitochondrial respiratory states and control indices were determined as described elsewhere^47,48^. OXPHOS flux was determined from the rate of ADP-stimulated mitochondrial respiration reflective of the ATP synthesis capacity and compared between the pre-and post-hypoxic conditions. Pilot studies showed that the LEAK respiration rate in control mitochondria (i.e. not exposed to H-R stress) was similar when measured in state 2 (with substrates but no ADP) and state 4 (in the presence of substrates, ADP and oligomycin) (Δ < 5%, *P* > 0.05). Therefore, both state 2 and state 4 respiration were considered representative of the mitochondrial proton leak reflecting the ETS activity needed to compensate for the futile proton and cation cycles in the absence of ATP synthesis^49^. Respiratory control ratio (RCR) was calculated as the ratio of OXPHOS to LEAK flux, and P-L control efficiency was calculated as the ratio of net to total OXPHOS capacity (1 − LEAK I + II/OXPHOS)^47,48^. To assess the rate of the electron leak, H_2_O_2_ efflux rate was divided by the oxygen consumption rate in the same cell isolate and expressed as H_2_O_2_ to O_2_ ratio.

### Quantitative real-time PCR

Total RNA was extracted from approximately 50 mg of tissue or isolated cells using TRIzol Reagent (Invitrogen) and the RNeasy Mini kit (Qiagen) with DNase I treatment according to the manufacturer’s recommendations. The quantity and quality of total RNA were determined using NanoDrop ND-2000 (Peqlab, Erlangen, Germany) and Bioanalyzer 2100 (Agilent Technologies, Waldbronn, Germany). The average tissue-specific RNA integrity number (RIN) was similar in all treatments (~ 6 in the muscle and ~ 8 in the brain).

For PCR analysis, 88 target genes and 43 miRNAs were selected (Supplementary Tables 1 and 2). The targets were chosen based on their important role in the HIF-1 regulation, apoptosis, redox homeostasis, glycolysis and mitochondrial OXPHOS^19^ and their previously reported association with hypoxia^31,50–52^. Due to the tissue-specific expression of the target transcripts, 67 mRNA transcripts and 41 miRNA were analyzed in the thalamus, and 41 mRNA transcripts and 34 miRNA were used in the masseter.

For cDNA synthesis, 200 ng of total RNA was mixed with 1 μL Reverse Transcription Master Mix (Fluidigm PN 100–6297) in 5 μL volume. The reaction was incubated at 25 °C for 5 min, 42 °C for 30 min followed by 85 °C for 5 min. The cDNA synthesis of miRNA was performed as described elsewhere^53^. In brief, 100 ng of total RNA were poly(A) tailed and reverse transcribed using 1 U poly(A)polymerase (BioLab), 0.1 mM of NTPs, RT-primers (CAGGTCCAGTTTTTTTTTTTTTTTVN where V is A, C or G and N is A, C, G or T) and 100 U MuLV reverse transcriptase (Invitrogen). The reaction was incubated at 42 °C for 1 h followed by 95 °C to inactivate the enzyme.

The cDNA obtained from mRNA and miRNA was used for qPCR with the Fluidigm BioMark HD System (Fluidigm Corporation, CA, USA). Specific target amplification (STA) was done per the manufacturer’s recommendations. Pre-amplification sample mixtures were prepared using PreAmp Master Mix (Fluidigm PN 1,005,581) containing 1.25 μl of cDNA, 1 μl PreAmp Master Mix, 0.5 μl Pooled Delta Gene Assay Mix (500 nM) containing DNA-suspension buffer and primers in 5 μl total volume. The pre-amplification reaction was incubated at 95 °C for 2 min, followed by 10 cycles at 95 °C for 15 s and 60 °C for 4 min. The pre-amplification reaction was cleaned up using exonuclease I and 10 × diluted with DNA suspension buffer (TEKnova, PN T0221). Fluidigm quantitative measurement runs were carried out with 96.96 dynamic arrays (Fluidigm Corporation, CA, USA) according to manufacturer’s instructions. In brief, 2.5 μl of 2 × SsoFast Evagreen Supermix with Low ROX, 0.25 μl 20 × sample-loading reagent, and 2.25 μl of treated samples were prepared. Separately, an assay mixture was prepared for each primer pair including 2.25 μl of DNA Suspension buffer, 0.25 μl of 100 μM forward and reverse primer (Supplementary Tables 1 and 2) and 2.5 μl of 2 × assay-loading reagents. The dynamic arrays were primed with control line fluid and loaded with the sample and assay mixtures via appropriate inlets using an IFC controller. The array chips were placed in the BioMark Instrument for PCR at 95 °C for 10 min, followed by 30 cycles at 95 °C for 15 s and 60 °C for 1 min. The data were analyzed with real-time PCR analysis software in the BioMark HD instrument (Fluidigm Corporation, San Francisco, CA). For normalization, the internal controls of cel-miR-39-3p, SSC_5S, SSC_Met_tRNA and SSC_U6 were used for miRNA and 5 housekeeping genes (GAPDH, U6, ACTB, RPL11 and RPL32) were used for mRNA (Supplementary Tables 1 and 2). Data analysis was done by 2 − ΔCt method. The sample size was 6 for all treatment groups.

### Data analysis

Data on mitochondrial functional traits (ṀO_2_ and ROS production) were tested for normality and homoscedasticity. All data were normally distributed, and most had equal variances except for two set of data before and after the H-R stress (ROS efflux in the LEAK I + II state of brain cells and H_2_O_2_ to O_2_ ratio in the OXPHOS state of muscle cells). We therefore used non-parametric tests for these two datasets. To test the tissue-specific variability in oxygen consumption rates and ROS production during different respiratory states (LEAKI, LEAKI + II, OXPHOS) under control (normoxic) conditions, a two-way ANOVA was conducted followed by Tukey's Honest Significant Difference (HSD) test. Tissue type and respiratory states were designated as between subject factors. The effects of hypoxia and reoxygenation on mitochondrial ṀO_2_ and ROS production were tested using paired two-tailed Student’s t-test or Wilcoxon test. All statistical analyses were conducted using IBM® SPSS® Statistics ver. 22.0.0.0 (IBM Corp., Armonk, NY, USA) and GraphPad Prism ver 7.02 (GraphPad Software Inc., La Jolla, CA, USA) software. Differences were considered significant if the probability of Type I error (*P*) was < 0.05.

mRNA and miRNA data were analyzed using SAS ver. 9.4. Treatment group (normoxia, hypoxia and reoxygenation) was used as a fixed effect. Samples were used as repeated measures factor using proc mixed procedure. We also compared mRNA and miRNA levels in the tissues immediately after collection (stored at − 80 °C) with the isolated cells from the same tissue samples. The means between the treatment groups were compared using the post hoc Tukey–Kramer test adjusting for multiple comparisons.

We have followed the recommendation of evidence-based language^54^ for describing our results. The following thresholds were used: *P* = 0.049–0.011 (moderate evidence), *P* = 0.01–0.001 (strong evidence), *P* < 0.001 (very strong evidence).

## Results

### Tissue-specific mitochondria functional traits and ROS production

**Figure 1:**
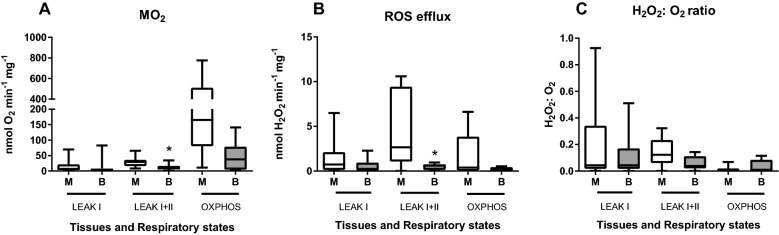
Baseline mitochondrial oxygen consumption (ṀO_2_) and ROS efflux rates in the permeabilized skeletal muscle and brain cells of *S. scrofa* under the control (normoxic) conditions. (**A**) oxygen consumption rates, (**B**) ROS efflux rates, (**C**) electron leak (the ratio of H_2_O_2_ produced to O_2_ consumed by the permeabilized cells). Tissues: M—skeletal muscle cells, B—brain cells. Mitochondrial traits were measured in non-phosphorylating mitochondria in the presence of Complex I substrates (LEAK I), the mixture of Complex I and II substrates (LEAKI+II), and in the phosphorylating (ADP-stimulated) mitochondria respiring on the mixture of the Complex I and II substrates (OXPHOS). Asterisks indicate significant differences in the respective traits between different tissues (*P*<0.05). N=6–7.

**Figure 2:**
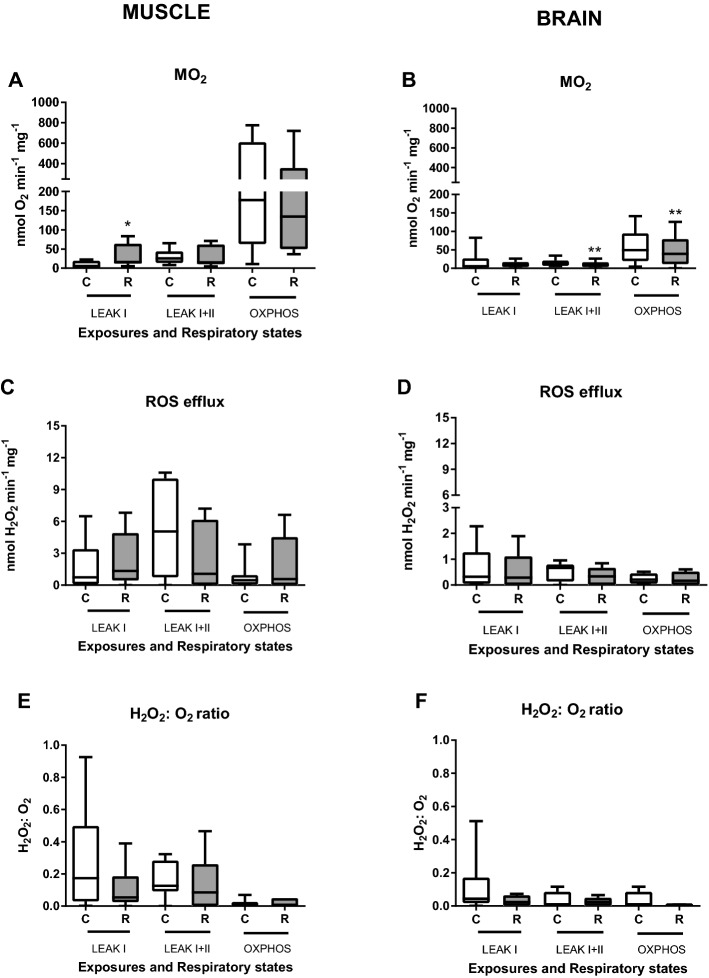
Effect of the H-R stress on the mitochondrial oxygen consumption (ṀO_2_) and ROS efflux rates in the permeabilized skeletal muscle and brain cells of *S. scrofa.* (**A**, **B**) oxygen consumption rates, (**C**, **D**) ROS efflux rates, (**E**, **F**) electron leak (the ratio of H_2_O_2_ produced to O_2_ consumed by the permeabilized cells). Conditions: C—normoxic (control) cells, R—cells after 15 min of severe hypoxia followed by 10 min of reoxygenation. Mitochondrial traits were measured in non-phosphorylating mitochondria in the presence of Complex I substrates (LEAK I), the mixture of Complex I and II substrates (LEAKI+II), and in the phosphorylating (ADP-stimulated) mitochondria respiring on the mixture of the Complex I and II substrates (OXPHOS). Asterisks indicate significant differences in the respective traits between the control and reoxygenation conditions (*P<0.05, **P<0.01). N=6–7 for the skeletal muscle and 6 for the brain.

Under normoxic conditions, mitochondria from the isolated porcine cells showed good coupling with the mean RCR of 8.8 and 4.2 respiring on the mixture of Complex I and II substrates. The mean P-L control efficiency with the combination of Complex I and II substrates was 0.88 and 0.76 in the muscle and the brain, respectively. ANOVA showed moderate evidence for the effects of the tissue type on the mitochondrial oxygen consumption and ROS production across three studied mitochondrial states (LEAK I, LEAK I + II and OXPHOS) in normoxia (Table 1). Pairwise comparisons of means showed moderate evidence (*P* < 0.05) for higher oxygen consumption rates (ṀO_2_) and ROS efflux during LEAK I + II respiration in the muscle relative to the brain cells (Fig. 1A,B). Although OXPHOS ṀO_2_ was considerably (~ 4–9-times) higher than LEAKI + II ṀO_2_, the ROS efflux rate was lower in the OXPHOS than LEAK state (Fig. 1B, Fig. 2). This was reflected in the lower electron leak (H_2_O_2_ to O_2_ ratio) in the OXPHOS relative to the LEAK state in both studied tissues (Fig. 1C).

**Table 1:** ANOVA: Effects of tissue type and respiratory states (LEAK I, LEAK I+II, OXPHOS) on the oxygen consumption rates (ṀO_2_), ROS efflux and H_2_O_2_:O_2_ ratio of permeabilized muscle and brain cells under the control (normoxic) conditions.

	Tissue	Respiratory state	Tissue × Respiratory state
ṀO_2_	*F*_1,39_=4.879, *P*=**0.033**	*F*_2,39_=6.895, *P*=**0.03**	*F*_2,39_=3.823, *P*=0.30
ROS efflux	*F*_1,39_=9.595, *P*=**0.004**	*F*_2,39_=2.052, *P*=0.142	*F*_2,39_=1.894, *P*=0.164
H_2_O_2_:O_2_ ratio	*F*_1,39_=1.251, *P*=0.270	*F*_2,39_=2.918, *P*=0.066	*F*_2,39_=0.553, *P*=0.580

*F* values with the degrees of freedom for the effect and error (in subscripts) are shown. Significant *P* values (*P*<0.05) highlighted in bold.

### Effects of H-R stress on mitochondria functional traits

**Figure 3:**
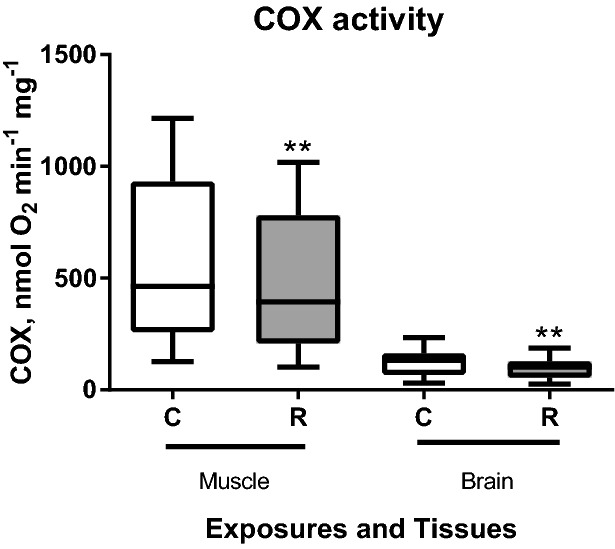
Effect of the H-R stress on the indices of mitochondrial coupling and cytochrome c oxidase (COX) activity in the permeabilized skeletal muscle and brain cells of *S. scrofa.* Conditions: C—normoxic (control) cells, R—cells after 15 min of severe hypoxia followed by 10 min of reoxygenation. Asterisks indicate significant differences in the respective traits between the control and reoxygenation conditions (**P<0.01). N=8 for the skeletal muscle and 7 for the brain.

There was no evidence of difference in the LEAK I + II or OXPHOS ṀO_2_ of the skeletal muscle cells under the control (normoxic) conditions and after 15 min acute exposure to severe hypoxia (*P* > 0.05)(Fig. 2A). However, muscle cell mitochondria showed moderate evidence of increase in LEAK I ṀO_2_ (with Complex I substrate) after exposure to H-R stress compared to the normoxic controls (*P* < 0.05). ROS production showed no evidence of change after H-R exposure in the muscle cells regardless of the mitochondrial state (*P* > 0.05) (Fig. 2C). In the brain cell mitochondria, LEAK I + II ṀO_2_ and OXPHOS ṀO_2_ with Complex I and II substrates showed strong evidence (*P* < 0.01) of suppression after H-R exposure (Fig. 2B). Similar to the muscle cells, there was no evidence for change in ROS production between normoxia and H-R exposure in the brain cells regardless of the mitochondrial state (*P* > 0.05) (Fig. 2D). Exposure to H-R stress had no effect on the RCR or P-L efficiency in the muscle and brain cells (*P* > 0.05, data not shown). COX activity showed strong evidence of decrease (*P* < 0.01) after reoxygenation in both studied tissues (Fig. 3).

### Expression of mRNA transcripts in brain and skeletal muscle tissue

Cell isolation had a significant effect on the transcript profiles of the muscle and the brain cells. In the masseter, 20 of 41 analyzed mRNA transcripts showed different expressions between the immediately frozen tissue and isolated cells with 14 transcripts showing higher and 6—lower levels in isolated cells than in the fresh tissue (Supplementary Table 3). In the thalamus, 50 of 67 analyzed transcripts showed significantly different expressions between the immediately frozen tissue and isolated cells with most (43) transcripts showing higher levels in isolated cells than the fresh tissue (Supplementary Table 4).

**Figure 4:**
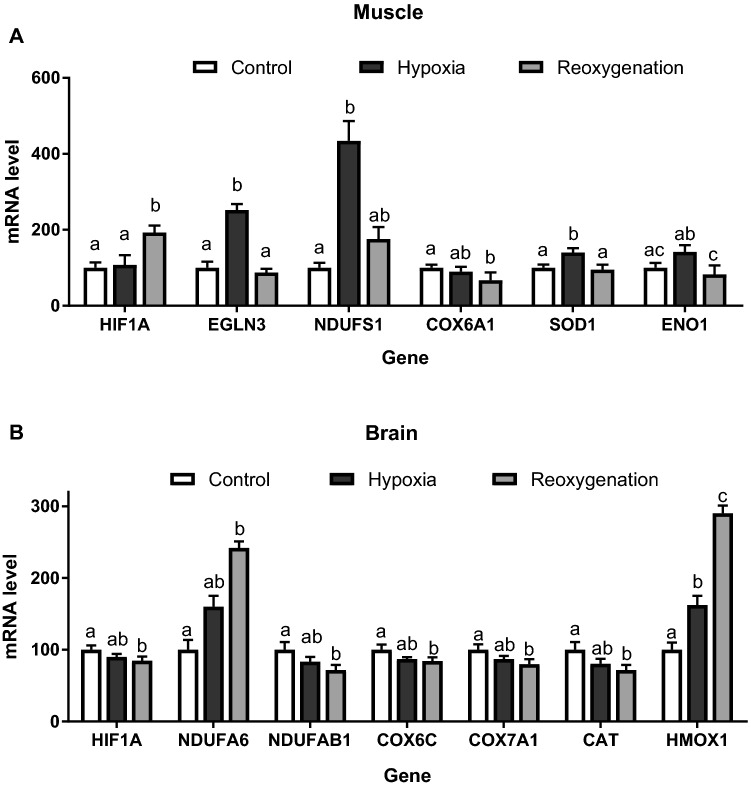
Effect of the H-R stress on mRNA expression of hypoxia-responsive genes in the isolated skeletal muscle and brain cells of *S. scrofa.* HIF1A—Hypoxia Inducible Factor 1 Alpha Subunit, EGLN3—Egl-9 Family Hypoxia Inducible Factor 3, NDUFS1—NADH: Ubiquinone Oxidoreductase Core Subunit S1, COX6A1- Cytochrome C Oxidase Subunit 6A1, SOD1- Superoxide Dismutase 1, HMOX1- Heme Oxygenase 1, ENO1- Enolase 1, NDUFA6—NADH: Ubiquinone Oxidoreductase Subunit A6, SDHC—Succinate Dehydrogenase Complex Subunit C, COX6C—Cytochrome c Oxidase Subunit 6C, COX7A1—Cytochrome C Oxidase Subunit 7A1, and CAT- Catalase. (**A**) skeletal muscle cells, (**B**) brain cells. Only the transcripts that showed significant (*P*<0.05) changes in hypoxia and/or reoxygenation relative to the normoxic controls are shown. Letters indicate differences between the exposure conditions; columns that do not share a letter are significantly different (*P*<0.05).

Acute short-term exposure of the isolated muscle cells to hypoxia and reoxygenation significantly altered expression levels of six mRNA transcripts (HIF1A, EGLN3, NDUFS1, COX6A1, SOD1 and ENO1). Three transcripts (EGLN3, NDUFS1 and SOD1) were significantly upregulated in the muscle cells exposed to 15 min of severe hypoxia and returned to the baseline (normoxic) conditions after 10 min of reoxygenation (Fig. 4A). Reoxygenation led to upregulation of HIF1A mRNA levels and a decrease in COX6A1 transcripts relative to the normoxic cells in the muscle cells (Fig. 4A). Transcript levels of ENO1 tended to increase in hypoxia and decrease during reoxygenation relative to the normoxic controls, but the differences in ENO1 mRNA levels were only statistically significant between the muscle cells exposed to hypoxia and those after reoxygenation (Fig. 4A).

Exposure of isolated thalamus cells to acute short-term hypoxia and reoxygenation led to a significant change in the transcript levels of seven mRNA (HIF1A, NDUFA6, NDUFAB1, COX6C, COX7A1, CAT and HMOX1). Hypoxia led to upregulation of HMOX1 transcript levels in the thalamus cells, and this increase was further enhanced by reoxygenation (Fig. 4B). Reoxygenation also led to upregulation of NDUFA6 mRNA. Levels of five transcripts (HIF1A, NDUFAB1, COX6C, COX7A1 and CAT) were not significantly affected by hypoxia, but decreased during post-hypoxic reoxygenation in the thalamus cells (Fig. 4B).

### Expression of miRNA transcripts in brain and skeletal muscle tissue

In the masseter, 7 of 34 analyzed miRNA showed significantly higher expression in the freshly collected tissue than in isolated cells (Supplementary Table 5). In the thalamus, 33 of 41 analyzed miRNA were differentially expressed in the isolated cells relative to the freshly collected tissue, with higher expression of 31 miRNA and lower expression of 2 miRNA in the isolated cells (Supplementary Table 6).

**Figure 5:**
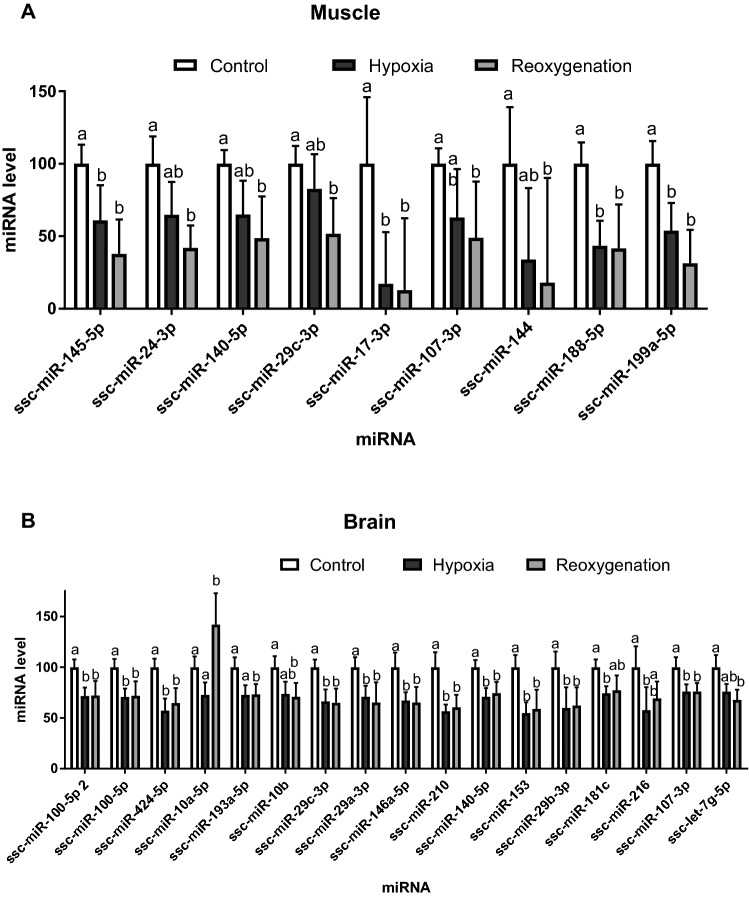
Effect of the H-R stress on miRNA expression in the isolated skeletal muscle and brain cells of *S. scrofa.* (**A**) skeletal muscle cells, (**B**) brain cells. Only the miRNAs that showed significant (*P*<0.05) changes in expression in hypoxia and/or reoxygenation relative to the normoxic controls are shown. Letters indicate differences between the exposure conditions; columns that do not share a letter are significantly different (*P*<0.05).

Of the studied 34 miRNAs in the masseter cells, 9 miRNAs (miR-145-5p, miR-24-3p, miR-140-5p, miR-29-3p, miR-17-3p, miR-107-3p, miR-144, miR-188-3p and miR-199a-3p) showed moderate to very strong evidence of altered expression in response to acute H-R stress (Fig. 5A). The levels of these differentially expressed miRNAs were suppressed in hypoxia and reoxygenation with generally stronger suppression after reoxygenation (Fig. 5A).

Of the 41 studied miRNAs in the thalamus cells, 17 miRNAs (ssc-miR-100-5p 2, ssc-miR-100-5p, ssc-miR-424-5p, ssc-miR-10a-5p, ssc-miR-193a-5p, ssc-miR-10b, ssc-miR-29c-3p, ssc-miR-29a-3p, ssc-miR-146a-5p, ssc-miR-210, ssc-miR-140-5p, ssc-miR-153, ssc-miR-29b-3p, ssc-miR-181c, ssc-miR-216, ssc-miR-107-3p, ssc-let-7 g-5p) showed moderate to very strong evidence of differential expression after acute H-R stress relative to the normoxic controls (Fig. 5B). Except for miR-10a-5p (significantly upregulated during post-hypoxic recovery), all other differentially expressed miRNAs were suppressed under hypoxia and/or reoxygenation in the brain cells (Fig. 5B).

## Discussion

### Effects of hypoxia-reoxygenation on mitochondrial activity and gene expression

Mitochondria of the muscle (masseter) cells of *S. scrofa* were more tolerant to the acute H-R stress than those of the brain (thalamus) cells. Thus, mitochondria of the brain cells showed suppressed mitochondrial respiration in the resting (LEAKI + II) state and a decrease in the OXPHOS (indicative of the maximum ATP synthesis capacity) following acute H-R stress. Furthermore, in three out of eight brain cell isolates, the oxygen consumption was not measurable after H-R stress indicating a complete loss of mitochondrial activity (data not shown). In contrast, mitochondria from the muscle cells showed stable LEAKI + II respiration and OXPHOS capacity following reoxygenation. This indicates that the muscle cells might be able to better restore ATP synthesis after exposure to short-term intermittent hypoxia than the brain cells consistent with the expected higher hypoxia tolerance of the muscle tissue^55^. Interestingly, in both studied cell types exposure to intermittent hypoxia led to the suppression of COX activity by 17–20% and was associated with a decrease in the transcript levels of genes encoding COX subunits (COX6A1 in the muscle and COX6C and COX7A1 in the brain). Possible mechanisms inhibiting COX activity (not tested in our present study) might also include cytochrome c release from the inner mitochondrial membrane^56,57^ or phosphorylation of serine or threonine residues on Complex IV subunits^58,59^. In mammals, COX activity can be modulated by the replacement of COX4-1 subunit through the hypoxia-specific COX4-2 subunit during hypoxia^60^. However, this replacement increases COX activity^60,61^ and thus cannot explain suppressed COX activity found in our present study. In porcine cells, COX shows large apparent excess capacity (~ 2.5–3 fold) relative to the OXPHOS activity, so the observed 17–20% decrease in the maximum COX velocity is unlikely to have a major effect on the ATP synthesis rates under most physiological conditions. However, a modest decrease in COX activity can play an important regulatory role^62^ and has been shown to attenuate H-R injury in mammals^63^. Interestingly, the porcine muscle cells showed elevated resting (LEAK I) respiration with pyruvate after H-R stress. Elevated rates of the pyruvate-driven respiration indicate activation of the forward flux of the electrons through mitochondrial Complex I and might represent a mechanism to mitigate ROS production by preventing the reverse electron transport (RET) caused by succinate accumulation in hypoxia^5^. This hypothesis is supported by the observation that succinate addition stimulated ROS efflux in the porcine muscle cells under normoxic conditions, but this increase was attenuated in the cells exposed to H-R stress. An increase in the Complex I activity in the muscle cells after reoxygenation was associated with a strong overexpression of NDUFS1 transcript encoding a key regulatory Complex I subunit^64,65^. NDUFS1 deficiency suppresses Complex I activity and increases ROS production in different types of mammalian cells^64,65^. Overexpression of NDUFS1 mRNA in hypoxia might thus represent an anticipatory response of the porcine muscle cells to support high Complex I activity and mitigate ROS during reoxygenation. In the mitochondria of the brain cells, hypoxia-reoxygenation stress had no effect on pyruvate-driven resting respiration. Interestingly, succinate addition did not stimulate ROS efflux in the brain mitochondria suggesting that RET might be a less important mechanism of ROS generation in the thalamus cells than in the muscle.

In the porcine brain cells, transcripts of two Complex I subunits, NDUFA6 and NDUFAB1, were differentially expressed in response to H-R stress. The mRNA levels of these subunits showed opposite directions of change during reoxygenation with suppression of NDUFAB1 and upregulation of NDUFA6 mRNA. NDUFAB1 subunit, like NDUFS1, plays an important role in regulating ETS flux and ROS production in mitochondria and its overexpression mitigates ischemia–reperfusion injury in mammalian cells^66^. Therefore, transcriptional downregulation of NDUFAB1 might be maladaptive during H-R and contribute to hypoxia sensitivity of the porcine brain cells. Unlike NDUFS1 and NDUFAB1, the role of NDUFA6 in the regulation of mitochondrial function is less well understood^67,68^.NDUFA6A is an accessory subunit involved in Complex I assembly^69^ and contains a site involved in the conversion of Complex I from active to inactive form (A/D conversion) during H-R^70^.Our present data do not permit inferences about the functional implications of the shift in NDUFA6 transcript levels, but the opposite direction of change of NDUFA6 and NDUFAB1 transcripts point out a possible dysregulation of Complex I in the thalamus cells during reoxygenation.

Various studies have reported on ROS production, its detrimental effects and relevance in both skeletal muscle and brain cells during H-R stress^71–73^. However, in our present study, there was no evidence of elevated ROS production during reoxygenation indicating that the observed differences in the mitochondrial response to H-R stress between the muscle and brain cells cannot be attributed to differences in the H-R-induced ROS burst.

### Transcriptional response of HIF-1 pathway and downstream HIF-1 targets

HIF-1 is a master transcriptional regulator of adaptive response to hypoxia in mammals^19^. HIFs are regulated post-translationally by oxygen-dependent hydroxylation of proline residues by prolyl hydroxylase domain protein (PHD) that targets HIF-1α for degradation^74^.The PDH activity is inhibited in hypoxia leading to the accumulation of HIF-1α and transcriptional activation of HIF-1 targets^75,76^. Our present study shows that HIF-1α and PHD3 (EGLN3) are regulated at the transcriptional level during H-R stress in the porcine cells. In our data, transcripts of three isoforms of PHD (EGLN1/PHD2, EGLN2/PHD1 and EGLN3/PHD3) as well as HIF-1α and HIF-2α subunits were found in the muscle and the brain cells of *S. scrofa*. However, only EGLN3 (in the muscle) and HIF-1α transcripts (in both studied cell types) showed evidence of the impact of H-R stress. EGLN3 transcripts were upregulated during hypoxia in porcine muscle cells, possibly to compensate for lower PHD activity caused by oxygen deprivation. Transcriptional upregulation of PHD3/EGLN3 has been shown to regulate the HIF response under low oxygen conditions ensuring hypoxic cell survival^77,78^. During reoxygenation, a decline in EGLN3 transcript levels was accompanied by upregulation of HIF-1α transcripts in the porcine muscle cells. An increase in HIF-1α levels promotes tolerance to H-R in a variety of mammalian systems^79–81^ and transcriptional upregulation of HIF-1α mRNA contributes to such increase^82^. Interestingly, a strong increase in HIF-1α expression was found during reoxygenation in an extremely hypoxia-tolerant marine invertebrate, the hard shell clam Mercenaria mercenaria suggesting the adaptive role of HIF-1 in post-hypoxic survival^83^ . In porcine brain cells, no transcriptional upregulation of EGLN3 or HIF-1α was found during hypoxia and reoxygenation. Furthermore, HIF-1α mRNA levels were suppressed during reoxygenation indicating that muted HIF-1 response might contribute to lower H-R tolerance of this cell type.

Upregulation of HIF-1 induces an adaptive switch to glycolysis in mammalian cells^40^.HIF-1 upregulates the transcription of multiple glycolytic enzymes such as enolase 1, aldolase, hexokinase and glyceraldehyde-3-phosphate dehydrogenase to enhance anaerobic ATP production under oxygen deficiency^74,84^. In the isolated porcine cells, all studied genes encoding glycolytic or glycogenolytic enzymes (including ENO1, PYGM, GPI, PGK1 and LDHB) showed stable transcript levels during H-R stress. This lack of glycolytic activation might be due to the short (15 min) hypoxia exposure that was insufficient to trigger anaerobic metabolism in the porcine cells in the present study.

Hypoxia has been reported to modulate antioxidant defense^85–87^. In our present study, out of the six studied antioxidant enzymes (heme oxygenase (HMOX1), superoxide dismutase (SOD), catalase (CAT), glutathione peroxidase, thioredoxin and copper chaperone for superoxide dismutase), three (CAT, SOD1 and HMOX1) were transcriptionally modulated by H-R exposure. In the muscle cells, SOD1 transcripts encoding cytosolic Cu, Zn-SOD were modestly and transiently upregulated during hypoxia returning to the baseline levels during reoxygenation. No other studied antioxidant transcripts responded to H-R stress in the isolated muscle cells. In the brain cells, HMOX1 was significantly overexpressed in both hypoxia and recovery, whereas CAT transcripts were suppressed during the post-hypoxic recovery in the brain cells. HMOX1 is associated with the degradation of heme and is involved in alleviating ischemic injury^86^. While the upregulation of HMOX1 transcripts might be considered protective, transcriptional suppression of CAT indicates dysregulation of antioxidant response during the post-hypoxic recovery of the brain cells. Taken together, these findings show that acute short-term H-R exposure did not induce strong oxidative stress consistent with the observation of the stable ROS emission during H-R stress in the isolated muscle and brain cells.

### Effects of hypoxia-reoxygenation on cellular miRNA profiles

MicroRNAs are important posttranscriptional regulators involved in the adaptive and maladaptive responses to hypoxia (ischemia) and reoxygenation^88–90^. In the porcine muscle cells, 4 of 34 studied miRNA were suppressed in hypoxia and 9 were suppressed in reoxygenation relative to the normoxic controls. This suppression implies transcriptional derepression of mRNA targets of respective miRNAs. Three hypoxamiRs suppressed during H-R in the muscle cells (ssc-miR-107-3p and ssc-miR-29b-3p regulating apoptosis and ssc-miR-140-5p regulating angiogenesis) also showed a decrease during reoxygenation in the brain cells indicating that they are a part of a general cellular response to H-R stress. In human pulmonary artery smooth muscle cells, hypoxia-mediated downregulation of miR-140-5p inhibits proliferation and promotes apoptosis by targeting and regulating DNA methyltransferase 1 (DNMT1) and SOD2 expression^91^. The miR-29 family also includes many important apoptosis regulators. Thus, downregulation of miR-29a-3p induces both pro- and anti-apoptotic functions in cells^92^ whereas upregulation of miR-29b-3p induces apoptosis and activates caspase 3 proteins^93^ . Hence, decreased expression of these miRNAs in our study might indicate stimulation of apoptosis-related pathways in the muscle and brain cells exposed to H-R stress.

In the muscle cells, hypoxamiRs downregulated by hypoxia involved ssc-miR-199a-5p controlling HIF1A expression^94^ , a regulator of angiogenesis and mitochondrial antioxidants (ssc-miR-17-3p)^95^ and two miRNAs regulating autophagy and apoptosis (ssc-miR-145-5p and ssc-miR-188-5p)^96–99^ . Suppression of miR-199a-5p protects against H-R stress in mammalian heart and brain cells^100–103^, attenuates apoptosis^104,105^), and contributes to higher aerobic capacity and tolerance to high-altitude hypoxia in humans^106^. Previous studies have associated lower levels of ssc-miR-17-3p with elevated mitochondrial enzyme activities in human colorectal cancer cells^95,107^ . If similar mechanisms exist in the porcine masseter cells, suppression of miR-199a-5p and ssc-miR-17-3p might partially explain the ability of these cells to maintain high aerobic capacity during H-R stress found in our present study. Furthermore, downregulation of miR-145 and ssc-miR-188-5p suppresses autophagy and cell death^96,108^ and might enhance post-hypoxic survival of the muscle cells.

In the porcine brain cells, substantially more (17 of 41 studied) miRNAs were suppressed after H-R stress. This indicates that the hypoxia-induced metabolic reorganization involving hypoxamiRs affects a larger swath of the cellular pathways in the brain than in the muscle cells. Unlike the muscle cells, H-R stress suppressed the expression of hypoxamiRs regulating key energy-conserving metabolic pathways such as mitochondrial ETS, TCA cycle (3 miRNAs) and glycolysis (3 miRNAs). These findings indicate that the brain cells engage compensatory mechanisms to increase ATP synthesis in response to H-R stress. Other suppressed hypoxamiRs in the brain involved those regulating apoptosis (5 miRNAs), angiogenesis (3 miRNAs) and inflammation (3 miRNAs). Upregulation of inflammatory pathways in the H-R exposed brain cells is consistent with the damage reflecting lower hypoxia tolerance of this cell type compared with the muscle cells. Overexpression of ssc-miR-424 and ssc-miR-210 stabilizes HIF1A and attenuates hypoxia-induced apoptosis, angiogenesis and stress response in mammalian cells^109,110^. Downregulation of these miRNAs found in both hypoxic and post-hypoxic brain cells might therefore negatively affect survival of the brain cells.

Interestingly, ssc-miR-10a-5p showed a different pattern of H-R-induced expression compared with other studied miRNAs increasing during the post-hypoxic recovery in the brain cells. miR10a-5p has been reported as a key regulator of tissue inflammation^111^. In stem and kidney cells downregulation of miR-10a-5p during hypoxia inhibits inflammatory responses, suppresses inflammatory gene expression and increases the proliferative ability of cells^112,113^. In contrast, in rat liver, miR-10a-5p was upregulated under hypoxic exposure and suggested to be responsible for regulating cell survival^111,114^. In primary murine adipocytes, elevated miR-10a5p facilitated cell cycle and interfered with fat deposition^115^. Given this controversy, the implications of the observed increase of ssc-miR-10a-5p during reoxygenation in the brain cells remain unclear and require further investigation.

### Conclusions, limitations and outlook

**Figure 6:**
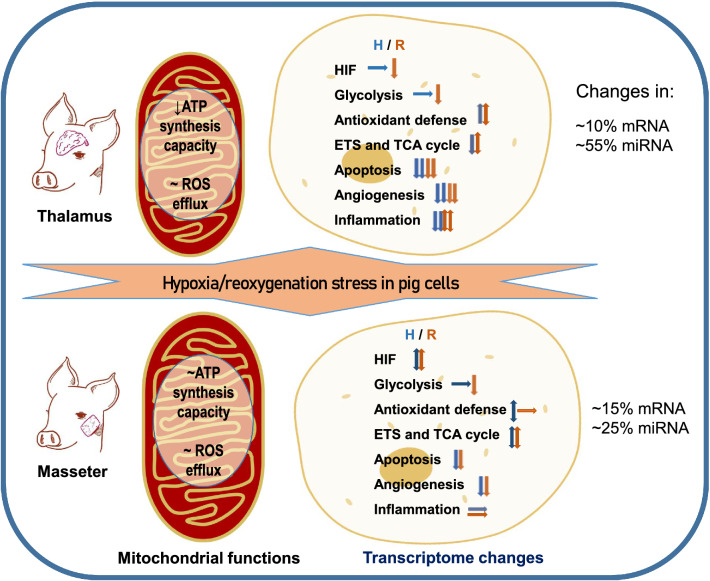
Summary of the observed mitochondrial and transcriptomic changes in response to acute H-R stress in the brain and the muscle cells of the pigs. Mitochondrial functions were assessed only after reoxygenation. mRna and miRNA profiles were measured after hypoxia (blue arrows) and reoxygenation (red arrows). Downward arrows show a decrease, upwards arrows—increase, horizontal arrows—no change. Single arrows—moderate change, double arrows—strong change.

The present study using isolated primary cells demonstrated that differences in mitochondrial tolerance and transcriptional regulation underlie the greater sensitivity of the brain cells to H-R stress compared with the skeletal muscle cells (Fig. 6). At the mitochondrial level, H-R stress led to a loss of the ATP synthesis capacity in the brain cells whereas in the skeletal muscle the ATP synthesis capacity was preserved and the forward flux through Complex I enhanced after short-term H-R exposure. The impaired mitochondrial aerobic capacity in the brain cells went hand-in-hand with the transcriptional upregulation of anaerobic glycolysis, likely as a compensatory mechanism to cover ATP deficiency. Notably, no elevated ROS efflux (above the respective tissue-specific baseline) was recorded in either cell type during the H-R stress showing that existing cellular antioxidants were sufficient to maintain the normal ROS levels during acute short-term H-R stress in isolated pig cells. Amplex UltraRed (used in the present study) is a highly sensitive probe for ROS and can detect low levels of H_2_O_2_ and (in the presence of extraneous SOD) superoxide. Superoxide and H_2_O_2_ are considered the main mitochondrial ROS in mammals^116^, but we cannot exclude production of other types of reactive oxygen species (such as singlet oxygen) during reoxygenation that cannot be detected by Amplex UltraRed. It is also worth noting that the present study used short-term (15 min) exposure of the cells to hypoxia followed by 10 min of reoxygenation, and longer exposures might induce stronger mitochondrial response and result in greater damage in the studied cell types.

The key elements of oxygen sensing (including HIF-1 and/or PHD) were transcriptionally modulated by H-R stress in both studied cell types, but the downstream effects differed between the brain and the skeletal muscle cells. In the muscle cells, mitochondrial ETS as well as protective mechanisms (including antioxidants and mitochondrial quality control) were transcriptionally unregulated, whereas in the brain cells apoptosis and inflammation pathways were the most responsive indicating cell damage. Although our results cannot be generalized for other tissues due to the tissue-specific differences in hypoxia response, we speculate that the transcriptionally regulation of the various pathways observed in our study might be applicable to other tissues with similar levels of hypoxia tolerance including sensitive (e.g. heart) or tolerant (e.g. vascular muscle, liver and kidney) tissues. These pathways can be further explored to fully understand the mechanisms associated with hypoxia tolerance across functionally different tissues. Furthermore, identification of the pathways modulated in the tolerant but not in the sensitive tissues might open avenues for future clinical interventions that mimic the tolerant molecular phenotype and desensitize tissues to H-R stress. Unexpectedly, our study showed transcriptional upregulation of multiple pathways related to metabolism, antioxidant defense and stress survival caused by the isolation of the muscle and brain cells from the respective tissues. This might be due to the improved nutrient and oxygen delivery to isolated cells suspended in the substrate-enriched media *ex situ*. These findings demonstrate that assessment of the transcriptional shifts between the tissues and isolated cells might serve as a sensitive marker for the deviation of the phenotype of isolated primary cells from the native *in situ* state and can be used in future studies as a measure of the preservation of the native metabolic phenotype during isolation of the primary cells.

## Supplementary Information

Supplementary Information.

## Data Availability

The data that supports the findings of this study are available in the supplementary material of this article. Additional data such as metadata can be obtained from the corresponding authors (mRNA and miRNA data: Dr. Siriluck Ponsuksili, ponsuksili@fbn-dummerstorf.de; mitochondrial data: Dr. Inna Sokolova inna.sokolova@uni-rostock.de) upon request.

## Abbreviations

ALDOC: Aldolase, fructose-bisphosphate C
ATP5B: ATP synthase F1 subunit beta
ATP5G2: ATP synthase membrane subunit C locus 2
ATP5J2: ATP synthase membrane subunit F
ATP5L: (ATP synthase membrane subunit G
ATP6V0D1: ATPase H + transporting V0 subunit D1
ATP6V1B2: ATPase H + transporting V1 subunit B2
ATP6V1C1: ATPase H + transporting V1 subunit C1
ATP6V1E1: ATPase H + transporting V1 subunit E1
ATP6V1F: ATPase H + transporting V1 subunit F
ATP5G1: ATP synthase membrane subunit C locus 1
CAT: Catalase
CCS: Copper chaperone for superoxide dismutase
COX10: Cytochrome C oxidase subunit 10
COX15: Cytochrome C oxidase subunit 15
COX4-1: Cytochrome C oxidase subunit 4 isoform 1
COX4-2: Cytochrome C oxidase subunit 4 isoform 2
COX5B: Cytochrome C oxidase subunit 5B
COX6A1: Cytochrome C oxidase subunit 6A1
COX6C: Cytochrome C oxidase subunit 6C
COX7A1: Cytochrome C oxidase subunit 7A1
COX7A2: Cytochrome C oxidase subunit 7A2
CS: Citrate synthase
DNMT1: DNA Methyltransferase 1
EGLN1: Egl-9 family hypoxia inducible factor 1
EGLN2: Egl-9 family hypoxia inducible factor 2
EGLN3: Egl-9 family hypoxia inducible factor 3
ENO1: Enolase 1
GPI: Glucose-6-phosphate isomerase
GPX3: Glutathione peroxidase 3
GPX4: Glutathione peroxidase 4
HIF1A: Hypoxia inducible factor 1 subunit alpha
HIF1AN: Hypoxia inducible factor 1 subunit alpha inhibitor
HIF2A: Hypoxia inducible factor 2 subunit alpha
HK2: Hexokinase 2
HMOX1: Heme oxygenase 1
LDHB: Lactate dehydrogenase B
MCL1: MCL1 apoptosis regulator, BCL2 family member
NDRG4: NDRG family member 4
NDUFA10: NADH dehydrogenase [ubiquinone] 1 alpha subcomplex subunit 10
NDUFA11: NADH dehydrogenase [ubiquinone] 1 alpha subcomplex subunit 11
NDUFA12: NADH dehydrogenase [ubiquinone] 1 alpha subcomplex subunit 12
NDUFA13: NADH dehydrogenase [ubiquinone] 1 alpha subcomplex subunit 13
NDUFA3: NADH dehydrogenase [ubiquinone] 1 alpha subcomplex subunit 3
NDUFA4: NADH dehydrogenase [ubiquinone] 1 alpha subcomplex subunit 4
NDUFA5: NADH dehydrogenase [ubiquinone] 1 alpha subcomplex subunit 5
NDUFA6: NADH dehydrogenase [ubiquinone] 1 alpha subcomplex subunit 6
NDUFA8: NADH dehydrogenase [ubiquinone] 1 alpha subcomplex subunit 8
NDUFA9: NADH dehydrogenase [ubiquinone] 1 alpha subcomplex subunit 9
NDUFAB1: NADH:ubiquinone oxidoreductase subunit AB1
NDUFB7: NADH dehydrogenase [Ubiquinone] 1 beta subcomplex subunit 7
NDUFB8: NADH dehydrogenase [Ubiquinone] 1 beta subcomplex subunit 8
NDUFS1: NADH:ubiquinone oxidoreductase core subunit S1
NDUFS5: NADH:ubiquinone oxidoreductase core subunit S5
NDUFS6: NADH:ubiquinone oxidoreductase core subunit S6
NDUFS7: NADH:ubiquinone oxidoreductase core subunit S7
NDUFS8: NADH:ubiquinone oxidoreductase core subunit S8
NDUFV1: NADH dehydrogenase (Ubiquinone) Flavoprotein 1
PDK1: Pyruvate dehydrogenase kinase 1
PFKM: Phosphofructokinase, muscle
PGC-1a: Peroxisome proliferator-activated receptor gamma coactivator 1-alpha
PGK1: Phosphoglycerate Kinase 1
PYGM: Glycogen phosphorylase, muscle associated
SDHA: Succinate dehydrogenase complex flavoprotein subunit A
SDHB: Succinate dehydrogenase complex flavoprotein subunit B
SDHC: Succinate dehydrogenase complex flavoprotein subunit C
SDHD: Succinate dehydrogenase complex flavoprotein subunit D
SOD1: Superoxide dismutase [Cu–Zn]
SOD2: Superoxide dismutase 2, mitochondrial
TXN: Thioredoxin
