# Supplementary material for: Effects of hypoxia and reoxygenation on mitochondrial functions and transcriptional profiles of isolated brain and muscle porcine cells
Source: Sci Rep. 2022 Nov 18;12:19881. doi: 10.1038/s41598-022-24386-0 (PMC9674649; doi:10.1038/s41598-022-24386-0)
Supplement: Supplementary file 1 — Supplementary Information. [file 41598_2022_24386_MOESM1_ESM.docx]

Supplementary Table1. The list of miRNA primers used in the study.

| miRNA | primers forward | primers reverse | Function |
| --- | --- | --- | --- |
| SSC_5S | AGGGTCGGGCCTGGTTAGTA | GCGGTCTCCCATCCAAGTA |  |
| SSC_Met_tRNA | CAGAGTGGCGCAGCGGAAGC | CGATCCATCGACCTCTGGGTTATG |  |
| SSC_U6 | GCTTCGGCAGCACATATACTAAAAT | CGCTTCACGAATTTGCGTGTCAT |  |
| cel-miR-39-3p | gtcaccgggtgtaaatcag | ggtccagtttttttttttttttcaag |  |
| ssc-let-7g-5p | cgcagtgaggtagtagtttg | ccagtttttttttttttttaactgcac | Apoptosis |
| ssc-miR-100-5p | cagaacccgtagatccgaact | gtccagtttttttttttttttcacaag | Glycolysis |
| ssc-miR-100-5p 2 | cagaacccgtagatccga | gtccagtttttttttttttttcacaag | Glycolysis |
| ssc-miR-107 | gcagagcagcattgtacag | ggtccagtttttttttttttttgatag | Apoptosis |
| ssc-miR-10a-5p | gcagtaccctgtagatccga | aggtccagtttttttttttttttacaa | Angiogenesis |
| ssc-miR-10b | cagtaccctgtagaaccga | aggtccagtttttttttttttttacaa | Apoptosis |
| ssc-miR-125a | gcagtccctgagaccct | ggtccagtttttttttttttttacagt | Apoptosis |
| ssc-miR-125b | gcagacaagtcaggctct | ggtccagtttttttttttttttaggt | Apoptosis |
| ssc-miR-128 | cacagtgaaccggtctc | caggtccagtttttttttttttttaaag | Redox homeostasis |
| ssc-miR-132b | cgcagtaacaatctaaagcca | gtccagtttttttttttttttcgac |  |
| ssc-miR-135 | cgcagtatggctttttattcct | ggtccagtttttttttttttttcaca | Angiogenesis |
| ssc-miR-140-5p | gcagagtggttttaccctatg | ggtccagtttttttttttttttctac | Angiogenesis |
| ssc-miR-144 | agcgcagtacagtatagatga | ggtccagtttttttttttttttgtacat | Apoptosis |
| ssc-miR-145-5p | gtccagttttcccaggaatc | ggtccagtttttttttttttttaagg | Apoptosis |
| ssc-miR-146a-5p | gcagtgagaactgaattcca | ggtccagtttttttttttttttaacc | Inflammation |
| ssc-miR-150 | ctcccaacccttgtacca | ggtccagtttttttttttttttcact | Apoptosis |
| ssc-miR-151-5p | agtcgaggagctcacag | ggtccagtttttttttttttttactaga |  |
| ssc-miR-153 | cgcagttgcatagtcaca | ggtccagtttttttttttttttcac | Angiogenesis |
| ssc-miR-155-5p | gcgcagttaatgctaattgtg | ccagtttttttttttttttcccctatc | Angiogenesis |
| ssc-miR-17-3p | ctgcagtgaaggcactt | ggtccagtttttttttttttttctaca | Mitochondria |
| ssc-miR-181c | gaacattcaacctgtcggt | ggtccagtttttttttttttttactca | Mitochondria |
| ssc-miR-182 | gtttggcaatggtagaactca | ggtccagtttttttttttttttagtgt | Angiogenesis |
| ssc-miR-184 | cagtggacggagaactga | gtccagtttttttttttttttaccct | Apoptosis |
| ssc-miR-188-5p | gcatcccttgcatggtg | gtccagtttttttttttttttaccct | Autophagy |
| ssc-miR-18a-5p | gcagtaaggtgcatctagtg | ggtccagtttttttttttttttatctg | HIF1 activity |
| ssc-miR-191 | caacggaatcccaaaagca | tccagtttttttttttttttcagct | HIF1 activity, apoptosis |
| ssc-miR-193a-5p | ggtctttgcgggcga | ggtccagtttttttttttttttcatct | Angiognesis |
| ssc-miR-199a-5p | gcccagtgttcagactac | gtccagtttttttttttttttgaacag | Glycolysis |
| ssc-miR-205 | ccttcattccaccggagt | ggtccagtttttttttttttttcaga | Apoptosis |
| ssc-miR-210 | gctgtgcgtgtgaca | gtttttttttttttttcagccgct | Mitochondria |
| ssc-miR-216 | tctcagctggcaactgt | ggtccagtttttttttttttttctca |  |
| ssc-miR-222 | GAGCTACATCTGGCTACTGG | GGTCCAGTTTTTTTTTTTTTTTGAG | Apoptosis |
| ssc-miR-24-3p | agtggctcagttcagca | gtccagtttttttttttttttctgttc | Apoptosis |
| ssc-miR-29a-3p | gcagctagcaccatctga | tccagtttttttttttttttaaccga | Apoptosis |
| ssc-miR-29b-3p | cagtagcaccatttgaaatcag | ggtccagtttttttttttttttaacac | Inflammation |
| ssc-miR-29c | gcagtagcaccatttgaaatc | aggtccagtttttttttttttttacc | Apoptosis, angiogenesis |
| ssc-miR-363 | agaattgcacggtatccatc | ggtccagtttttttttttttttacag | Apoptosis |
| ssc-miR-424-5p | agcagcagcaattcatgt | aggtccagtttttttttttttttcaa | Mitochondrial |
| ssc-miR-429-5p | gcagtaatactgtctggtaatgc | ggtccagtttttttttttttttacg | Calcuim regulation |
| ssc-miR-885-3p | gcagcggggtgtagt | ggtccagtttttttttttttttatcca | Glycolysis |
| ssc-miR-92b-5p | cgcagtattgcactcgtc | ccagtttttttttttttttggagtc | Proliferation |

Supplementary Table2. The list of mRNA primers used in the study.

| Genes | primers forward | primers reverse | Function |
| --- | --- | --- | --- |
| ALDOC | CATTGGATGGGAGGATAGGG | GAGAGCCCAAGGATAAGCAA | Glycolysis / Gluconeogenesis |
| ATP5B | TATGATGTTGCCCGTGGAGT | AACCTGGAATGGCTGAGACA | ATP synthase |
| ATP5G2 | ACTGTGTTTGGGAGCCTCAT | TCACATGGCGAAGAGGATGA | ATP synthase |
| ATP5J2 | CCGGAGCATTTCAAAGAGGT | GCTCCTTGTAAGAACGGCAG | ATP synthase |
| ATP5L | ATAACTCTCTTGCAGCCCGT | CCTGCTGGTGAATTTGGGAT | ATP synthase |
| ATP6V0D1 | CCGATTCTTTGAGCACGAGG | GCAATCCACACAATGTTGCG | V-ATPase |
| ATP6V1B2 | TTATCACTCCTCGCTTGGCT | GCTGCTGAAACCTCTCGAAG | V-ATPase |
| ATP6V1C1 | TGTACGGTGGCTGAAAGTGA | TTGGGTTGAAGGAGCATTGC | V-ATPase |
| ATP6V1E1 | ATACCTTAGAGAGCCGGCTG | GCGGGTGTCTTCAAATGTCA | V-ATPase |
| ATP6V1F | CTTTCCGGCAGTTCCTCAAC | TCCTTGGCAGCATCATAGGG | V-ATPase |
| ATP5G1 | GGAACAGTGTTTGGCAGCAT | GAGGATAAGGAAGGCGACCA | ATP synthase |
| CAT | AAAAACCTTGGCAAAATGAA | TTCAAAAGACCCCAAAGCA | Carbon metabolism |
| CCS | TCGATGAGGGAGAAGACGA | CTTGGGGTTCTGGAAGAGG | Interact with SOD1 |
| COX10 | TCTGGAAGGGTCTCTCTCGA | CCTTCTCCTTTGGCCTCAGA | Complex IV |
| COX15 | TTTGAGAATCCCACCACGGT | TAATTCCCAAGCCCACCTGT | Complex IV |
| COX5B | GGAGAGGGAGGTCATGATGG | TTCACAGATGCAGCCCACTA | Complex IV |
| COX6A1 | CCATCTTCGCATCAGGTCCA | GGGTAGTGGTCCAGGTTCTC | Complex IV |
| COX6C | TTGACCAAACCTCAGATGCG | TGCCTTCTTTCTTGGTTCAGC | Complex IV |
| COX7A1 | ACATCCTGTACCGAGTGACT | GCTTGTTGAGGTCCTGCAGA | Complex IV |
| COX7A2 | TGCATCTGAAGGGAGGGATC | CTAACTGAGTGCTGGGAGGA | Complex IV |
| CS | TTTGCTCTGAAACACCTGCC | AGTTCATCTCCGTCATGCCA | Carbon metabolism |
| EGLN1 | CAGCAATACCCCACTTCACC | TCATGCAGTACAAAGCCACA | HIF-1 signaling pathway |
| EGLN2 | GGCTGCTCATTTTCTGGTCT | GGACACCTTTCTGTCCTGCT | HIF-1 signaling pathway |
| EGLN3 | GACTGTCCCAGAAGGAAGCA | GGAAGGATGCAAGACAATGAA | HIF-1 signaling pathway |
| ENO1 | CTCCCCTCCCTGTGATGTCT | ACTTGGTGGAAGCGAGGTG | Glycolysis/Gluconeogenesis |
| GPI | CTGGTCCTTCTTGTCCCTTC | ACCGAAACACTTCAGCCAAC | Glycolysis/Gluconeogenesis |
| GPX3 | CAAGGCAGATGTGAGAGCAA | ACATCCCCCGAAGAAGCA | Redox homeostasis |
| GPX4 | CCCCAGGTCATAGAGAAGGA | GGGCTGGTTTTTAGGCAGA | Redox homeostasis |
| HIF1A | CAGCACGACTTGATTTTCTCC | GGCCAACAAAGTTAAAGCATC | HIF-1 signaling pathway |
| HIF1AN | TCTCTTCGGCTTCCCTCTAAC | AGAAAGCTTCCAAGCCCAGA | Cellular response to hypoxia |
| HIF2A | ACCGTCAACCTCAAGTCAGC | TTCTGTCGTCGCAGTAGGTG | Cellular response to hypoxia |
| HK2 | ATTGAACTGGGTTTTGCCTCT | ACGGGGATTAGCAAAGGTTC | Glycolysis/Gluconeogenesis |
| HMOX1 | TTTCTGAGCCTCCAAACACC | GACGGAAACACGAGACACAA | Redox homeostasis /HIF-1 signaling pathway |
| LDHB | GCTTGTTCCTTCAGACACCC | TGCACCAGATTGAGACGACT | Glycolytic enzyme activity |
| MCL1 | CCAAAATGGAAAGGGAGGA | GTGTTCACCCCCAACAGAA | Apoptosis |
| NDRG4 | GATAGCGCTGTGAATCTTGG | AAAACCACAGGCAGGAGGA | Apoptosis |
| NDUFA10 | GCCGAAGATGCAGAAAAGGT | CGGGAGATAGACGGGAATGG | Complex I |
| NDUFA11 | CAAGGACAGGACGGTACACA | CGCAGCCTCCAATGAAGTAG | Complex I |
| NDUFA12 | GCATCGTTGGCTTCACTGTA | GGTACGTACTGCTGTGGAGT | Complex I |
| NDUFA13 | ACTTGGAGGAGGAGGCAATC | TAGGTGGCGCTGAGAATCTC | Complex I |
| NDUFA3 | CCACACCCTACAACTACCCA | GAGGTGCTCACAGGTTCTTC | Complex I |
| NDUFA4 | GATCATCACGCAGGCCAAG | TGGGTTATTCTTCCTGTCCCA | Complex I |
| NDUFA5 | TGGTAATAGGGAGTTTGAGTCAG | TCACCTTTCAAATGCAGCTTCT | Complex I |
| NDUFA6 | CGATGCTGGATGGCTTTGTT | GCTGCTCTGATCCTTCTCCA | Complex I |
| NDUFA8 | TCTGAAGCCTGCCAAACATG | TGATCCCGGAAAGAACCCTC | Complex I |
| NDUFA9 | TCCCTTACCCTTTGCCACAT | GCCTGAATGCCAAGGTCTTC | Complex I |
| NDUFAB1 | CTGTGTCGCCGGTATAGTGA | GGTCCAAACTGTCTAAGCCC | Complex I |
| NDUFB7 | CTACCTCATCCAGCTGCTCA | TCCCGCTCAAACTCCTTCAT | Complex I |
| NDUFB8 | AGCAAGAGAGGGATCCATGG | TCCAAGAAACAGGAGTCGGG | Complex I |
| NDUFS1 | GCTGATCCACTTGTTCCACC | AGCAAAACTGGGTCCTGGTA | Complex I |
| NDUFS5 | ACGTCTGAGTGCCATCAAGA | CAGGGAAGAGGGATGGTGAA | Complex I |
| NDUFS6 | CGTCAGAAGGAGGTGAATGAG | CGTCTTCGTTTCCTTGTCCA | Complex I |
| NDUFS7 | TCTCCAAACCCACCACACTT | CAAAGCGGTCCATGTCGTAG | Complex I |
| NDUFS8 | TCGACATGACCAAGTGCATC | TGTTGTACAGCAGCTCTTCG | Complex I |
| NDUFV1 | CCGCCTCATCGAGTTCTACA | GTGGCCTTCTATCTGCTTGC | Complex I |
| PDK1 | GGAAGCATTACAACACCAACC | TTCTGAACACCATCCCTTCC | mitochondrial multienzyme complex |
| PFKM | AGAGGGCCTTGGTCTTTCAA | CCGAGTGATGTGCTCCAGAT | Glycolytic enzyme activity |
| PGC-1a | AGAGTATGAGAAGCGGGAGTC | CTCAGTTCTGTCCGTGTTGTG | Regulate OXHPOS |
| PGK1 | CCCTGGATTTGCCTATATTCCT | TTGTTTCCATGTTGAGTGGTG | Glycolytic enzyme activity |
| PYGM | AGTCTGTTCGCAATCCCTCA | TGAACACTGGGATCTGGAGG | Glycolytic enzyme activity |
| SDHA | GCAGAACCGAAGATGGCAAG | GCAGAGACCTTCCGTACAAC | Complex II |
| SDHB | TCCTATGGTGTTGGATGCGT | GTGTTGCCTCCGTTGATGTT | Complex II |
| SDHC | CCGACACTTGATTTGGGACC | ACTCAGCTCTTCACATGGCT | Complex II |
| SDHD | TCGTTACTGACTACGTGCGA | TGCAGATCCCCACATCATGA | Complex II |
| SOD1 | ACTGCTGGCAAAGATGGTGT | TCTGCCCAAGTCATCTGGTT | Antioxidant enzymes |
| SOD2 | CCCAGGGTTAACACTATCCA | TTGAGGACAAAATTTCACAAAAG | Antioxidant enzymes |
| TXN | TGTGGATGACTGTCAGGATGTT | TGGTGGCTTCAAGTTTTTCC | redox reactions |
| U6 | CTTCGGCAGCACACACTCTA | AGGGGCCATGCTAATCTTCT | house keeping |
| ACTB | GAGAAGCTCTGCTACGTCGC | CCTGATGTCCACGTCGCACT | house keeping |
| RPS11 | GAAACTGGCAAGGAGAAG | TTCGGATGTAGTGGAGGTAG | house keeping |
| RPL32 | AGCCCAAGATCGTCAAAAAG | TGTTGCTCCCATAACCAATG | house keeping |
| GAPDH | ATGCCTCCTGTACCACCAAC | AAGCAGGGATGATGTTCTGG | house keeping |

Supplementary Table 3. Differentially expressed mRNAs between the freshly dissected muscle tissue and muscle cell isolates

| Gene | Mean ± SE  Tissue | Mean ± SE Isolated cells | P-Value for difference |
| --- | --- | --- | --- |
| HIF1A | 0.022 ± 0.005 | 0.767 ± 0.108 | 0.008071407 |
| SOD1 | 2.126 ± 0.319 | 4.361 ± 0.388 | 0.006028852 |
| COX6A1 | 0.450 ± 0.037 | 3.202 ± 0.263 | 5.97217E-06 |
| NDUFA13 | 8.505 ± 0.576 | 3.771 ± 0.744 | 0.007128954 |
| PYGM | 7.384 ± 0.622 | 0.725 ± 0.111 | 2.26415E-06 |
| NDUFA9 | 4.608 ± 0.344 | 1.688 ± 0.485 | 0.007356472 |
| NDUFB7 | 4.024 ± 0.572 | 0.944 ± 0.233 | 0.000133712 |
| COX5B | 22.684 ±2.253 | 7.634 ± 1.137 | 0.002040544 |
| CS | 5.902 ± 1.085 | 1.614 ± 0.155 | 0.006762965 |
| ATP5B | 25.927 ± 2.969 | 5.980 ± 0.645 | 0.000663573 |
| NDUFV1 | 4.467 ± 0.725 | 0.577 ± 0.157 | 7.92719E-06 |
| ATPG1 | 20.525 ± 2.457 | 4.079 ± 0.765 | 4.02497E-05 |
| GPI | 3.319 ± 0.438 | 1.709 ± 0.483 | 0.040767935 |
| EGLN1 | 0.377 ± 0.087 | 1.931 ± 0.277 | 0.000347289 |
| NDUFB8 | 3.619 ± 0.166 | 1.501 ± 0.246 | 0.021465795 |
| NDUFS8 | 1.354 ± 0.181 | 0.342 ± 0.064 | 6.51793E-05 |
| LDHB | 2.726 ± 0.621 | 1.350 ± 0.164 | 0.002160587 |
| ATP5G2 | 7.992 ± 0.870 | 3.090 ± 0.586 | 0.000764805 |
| TXN | 1.066 ± 0.173 | 8.968 ± 1.022 | 1.00851E-06 |
| NDUFA8 | 0.427 ± 0.040 | 0.058 ± 0.015 | 0.044654603 |

Supplementary Table 4. Differentially expressed mRNAs between the freshly dissected brain tissue and brain cell isolates.

| Gene | Mean ± SE Tissue | Mean ± SE Isolated cells | P-Value for difference |
| --- | --- | --- | --- |
| ALDOC | 1.762 ± 0.104 | 4.027 ± 0.586 | 4.01786E-05 |
| ATP5B | 3.686 ± 0.291 | 12.285 ± 0.945 | 4.09366E-10 |
| ATP5G2 | 1.522 ± 0.084 | 2.136 ± 0.233 | 0.006116701 |
| ATP5J2 | 5.956 ± 0.349 | 8.473 ± 1.168 | 0.013076743 |
| ATP5L | 0.668 ± 0.030 | 0.462 ± 0.09 | 0.023056417 |
| ATP6V0D1 | 0.177 ± 0.007 | 1.279 ± 0.313 | 7.42459E-05 |
| ATP6V1B2 | 0.323 ± 0.026 | 1.392 ± 0.248 | 3.38791E-05 |
| ATP6V1E1 | 1.306 ± 0.081 | 3.132 ± 0.461 | 3.38791E-05 |
| ATP6V1F | 0.093 ± 0.013 | 0.936 ± 0.357 | 0.003317165 |
| ATPG1 | 1.749 ± 0.112 | 4.580 ± 0.931 | 0.00042786 |
| CAT | 0.191 ± 0.021 | 0.074 ± 0.02 | 4.61692E-05 |
| CCS | 0.007 ± 0.001 | 0.025 ± 0.003 | 1.76816E-06 |
| COX5B | 5.047 ± 0.186 | 10.736 ± 2.090 | 0.001285971 |
| COX6A1 | 5.044 ± 0.297 | 8.376 ± 1.070 | 0.000669432 |
| COX6C | 2.540 ± 0.189 | 1.761 ± 0.121 | 0.000383768 |
| COX7A1 | 0.836 ± 0.065 | 0.368 ± 0.067 | 5.72675E-06 |
| COX7A2 | 1.617 ± 0.098 | 2.108 ± 0.196 | 0.010021615 |
| COX10 | 0.001 ± 0.0004 | 0.016 ± 0.003 | 3.82855E-07 |
| COX15 | 0.0187 ± 0.004 | 0.049 ± 0.002 | 3.11271E-08 |
| CS | 1.364 ± 0.057 | 2.228 ± 0.348 | 0.002793669 |
| EGLN1 | 1.887 ± 0.082 | 0.731 ± 0.132 | 1.5515E-07 |
| EGLN2 | 0.020 ± 0.005 | 0.047 ± 0.008 | 0.001581742 |
| EGLN3 | 0.061 ± 0.002 | 0.140 ± 0.025 | 0.000334403 |
| ENO1 | 5.975 ± 0.342 | 8.688 ± 1.415 | 0.019843321 |
| GPI | 0.947 ± 0.050 | 3.798 ± 0.478 | 6.33545E-08 |
| GPX4 | 2.137 ± 0.125 | 6.235 ± 1.002 | 1.4743E-05 |
| HIF1A | 0.873 ± 0.052 | 0.140 ± 0.025 | 2.67433E-11 |
| LDHB | 2.268 ± 0.053 | 3.207 ± 0.549 | 0.036138639 |
| NDRG4 | 3.916 ± 0.235 | 28.312 ± 2.223 | 1.60637E-12 |
| NDUFA3 | 0.022 ± 0.002 | 0.081 ± 0.022 | 0.001353392 |
| NDUFA5 | 0.622 ± 0.03 | 0.332 ± 0.074 | 0.000591702 |
| NDUFA6 | 0.002 ± 0.0003 | 0.006 ± 0.001 | 0.008631802 |
| NDUFA8 | 0.049 ± 0.007 | 0.112 ± 0.012 | 5.68603E-06 |
| NDUFA9 | 0.718 ± 0.044 | 1.601 ± 0.1 | 2.31994E-09 |
| NDUFA10 | 0.128 ± 0.006 | 0.426 ± 0.101 | 0.000523457 |
| NDUFA11 | 0.430 ± 0.022 | 2.193 ± 0.565 | 0.000280601 |
| NDUFA13 | 2.842 ± 0.207 | 4.505 ± 0.471 | 0.00067583 |
| NDUFAB1 | 0.836 ± 0.084 | 1.714 ± 0.110 | 9.61331E-08 |
| NDUFB7 | 0.494 ± 0.035 | 1.697 ± 0.430 | 0.000843524 |
| NDUFB8 | 1.036 ±0.068 | 1.901 ± 0.143 | 1.93865E-06 |
| NDUFS6 | 0.016 ± 0.003 | 0.077 ± 0.021 | 0.000777537 |
| NDUFS7 | 0.003 ± 0.001 | 0.033 ± 0.012 | 0.002718485 |
| NDUFS8 | 0.125 ± 0.009 | 0.243 ± 0.052 | 0.005472019 |
| NDUFV1 | 0.367 ± 0.023 | 1.396 ± 0.33 | 0.000284175 |
| PDK1 | 0.124 ± 0.01 | 0.038 ± 0.008 | 2.13261E-05 |
| PFKM | 1.640 ± 0.1 | 2.730 ± 0.304 | 0.000418247 |
| PGC | 0.063 ± 0.007 | 0.042 ±0.008 | 0.020902289 |
| SDHA | 0.001 ± 0.0001 | 0.005 ± 0.002 | 0.00165176 |
| SDHC | 0.248 ± 0.012 | 0.331 ± 0.087 | 0.001497491 |
| SOD1 | 6.997 ± 0.312 | 9.057 ± 1.164 | 0.036599254 |
| ALDOC | 1.762 ± 0.104 | 4.027 ± 0.586 | 4.01786E-05 |
| ATP5B | 3.686 ± 0.291 | 12.285 ± 0.945 | 4.09366E-10 |
| ATP5G2 | 1.522 ± 0.084 | 2.136 ± 0.233 | 0.006116701 |
| ATP5J2 | 5.956 ± 0.349 | 8.473 ± 1.168 | 0.013076743 |

Supplementary Table 5. Differentially expressed miRNA between the freshly dissected muscle tissue and muscle cell isolates

| miRNA | Mean ± SE  Tissue | Mean ± SE Isolated cells | P-Value for difference |
| --- | --- | --- | --- |
| ssc-miR-191-5p | 1.214 ± 0.164 | 7.400 ± 1.018 | 0.011118446 |
| ssc-miR-125a-5p | 0.278 ± 0.034 | 0.892 ± 0.104 | 0.010314267 |
| ssc-miR-18a-5p | 1.215 ± 0.207 | 15.578 ± 3.046 | 0.01878789 |
| ssc-miR-199a-5p | 28.491 ± 4.459 | 57.257 ± 8.958 | 0.003613185 |
| ssc-miR-205 | 0.078 ± 0.025 | 0.423 ± 0.163 | 0.036536705 |
| ssc-miR-107-3p | 4.104 ± 0.676 | 10.831 ± 1.153 | 0.009999134 |
| ssc-miR-144 | 0.021 ± 0.006 | 1.368 ± 0.534 | 0.006662111 |

Supplementary Table 6. Differentially expressed miRNA between the freshly dissected brain tissue and brain cell isolates

| miRNA | Mean ± SE  Tissue | Mean ± SE Isolated cells | P-Value for difference |
| --- | --- | --- | --- |
| ssc-miR-222a | 0.617 ± 0.138 | 2.840 ± 0.313 | 2.63418E-06 |
| ssc-miR-363 | 4.224 ± 0.657 | 1.488 ± 0.211 | 3.79563E-05 |
| ssc-miR-145-5p | 0.495 ± 0.073 | 0.117 ± 0.027 | 5.73592E-07 |
| ssc-miR-125a-5p | 0.986 ± 0.134 | 4.673 ± 0.367 | 5.00629E-07 |
| ssc-miR-151-5p | 27.488 ± 3.510 | 79.173 ± 4.575 | 4.56435E-07 |
| ssc-miR-100-5p 2 | 88.215 ± 11.830 | 474.708 ± 36.738 | 1.28122E-07 |
| ssc-miR-191-5p | 2.103 ± 0.242 | 16.880 ± 1.988 | 2.41953E-06 |
| ssc-miR-100-5p | 79.859 ± 10.881 | 429.222 ± 35.111 | 1.46523E-07 |
| ssc-miR-125b-3p | 0.693 ± 0.116 | 1.824 ± 0.125 | 5.6268E-06 |
| ssc-miR-150-5p | 3.084 ± 0.511 | 8.648 ± 1.445 | 0.003407055 |
| ssc-miR-128 | 106.449 ± 26.077 | 323.518 ± 26.768 | 6.73915E-06 |
| ssc-miR-424-5p | 106.030 ± 16.742 | 442.509 ± 37.564 | 5.73592E-07 |
| ssc-miR-132b-3p | 0.0297 ± 0.007 | 0.128 ± 0.013 | 0.000224177 |
| ssc-miR-885-3p | 0.0316 ± 0.005 | 0.090 ± 0.003 | 1.93824E-05 |
| ssc-miR-10a-5p | 4.696 ± 0.725 | 24.742 ± 2.643 | 1.20978E-06 |
| ssc-miR-193a-5p | 0.038 ± 0.006 | 0.118 ± 0.011 | 3.27505E-06 |
| ssc-miR-181c | 7.758 ± 1.310 | 21.821 ± 1.688 | 1.30072E-05 |
| ssc-miR-10b | 0.127 ± 0.027 | 0.405 ± 0.044 | 3.38737E-05 |
| ssc-miR-92b-5p | 0.014 ± 0.003 | 0.071 ± 0.013 | 0.000279658 |
| ssc-miR-29c-3p | 100.042 ± 12.541 | 301.096 ± 22.773 | 3.10486E-06 |
| ssc-miR-24-3p | 22.977 ± 3.493 | 44.506 ± 3.017 | 0.000418754 |
| ssc-miR-29a-3p | 3.922 ± 0.580 | 12.641 ± 1.258 | 4.18709E-05 |
| ssc-miR-146a-5p | 0.052 ± 0.008 | 0.171 ± 0.024 | 4.78587E-05 |
| ssc-miR-107-3p | 45.247 ± 8.122 | 87.212 ± 8.597 | 0.000373695 |
| ssc-miR-184 | 0.113 ± 0.022 | 0.259 ± 0.053 | 0.019517464 |
| ssc-miR-210 | 1.605 ± 0.216 | 3.882 ± 0.578 | 0.000167696 |
| ssc-miR-135 | 20.359 ± 6.386 | 77.793 ± 26.137 | 0.018037498 |
| ssc-miR-18a-5p | 4.841 ± 0.631 | 10.983 ± 2.272 | 0.005168254 |
| ssc-miR-140-5p | 7.379 ± 0.988 | 12.209 ± 0.877 | 0.001255477 |
| ssc-miR-153 | 0.022 ± 0.004 | 0.050 ± 0.006 | 0.000484369 |
| ssc-miR-29b-3p | 80.748 ± 12.313 | 183.002 ± 28.150 | 0.003112408 |
| ssc-miR-144 | 0.013 ± 0.002 | 0.316 ± 0.134 | 0.010958965 |
| ssc-miR-216 | 0.129 ± 0.019 | 0.233 ± 0.048 | 0.037379138 |
| ssc-miR-222a | 0.617 ± 0.138 | 2.840 ± 0.313 | 2.63418E-06 |
| ssc-miR-363 | 4.224 ± 0.657 | 1.488 ± 0.211 | 3.79563E-05 |
